# Comparing presentations and outcomes of children with cancer: a study between a lower-middle-income country and a high-income country

**DOI:** 10.1186/s12887-023-04214-8

**Published:** 2023-09-05

**Authors:** Ahmed Farrag, Mohamed Hamdy Ghazaly, Khaled Mohammed, Ruth Volland, Barbara Hero, Frank Berthold

**Affiliations:** 1 Department of Pediatric Oncology, South Egypt Cancer Institute, Assiut University, Assiut, Egypt; 2 Division of Pediatric Hematology and Oncology, Department of Pediatrics and Adolescent Medicine, University Hospital of Cologne, Cologne, Germany; 3 Department of Pediatrics, Faculty of Medicine, Assiut University, Assiut, Egypt; 4 Department of Pediatrics and Adolescent Medicine, Mayo Clinic, Rochester, MN USA; 5 Division of Pediatric Hematology and Oncology, Department of Pediatrics and Adolescent Medicine, Statistics, University Hospital of Cologne, Cologne, Germany

**Keywords:** Cancer, Children, Lower-middle income country, Problems, Survival

## Abstract

**Background:**

Substantial progress has been achieved in managing childhood cancers in many high-income countries (HICs). In contrast, survival rates in lower-middle-income countries (LMICs) are less favorable. Here, we aimed to compare outcomes and associated factors between two large institutions; Egypt (LMIC) and Germany (HIC).

**Methods:**

A retrospective review was conducted on newly diagnosed children with cancer between 2006 and 2010 in the departments of pediatric oncology at the South Egypt Cancer Institute (SECI) (*n* = 502) and the University Hospital of Cologne-Uniklinik Köln (UKK) (*n* = 238). Characteristics including age, sex, diagnosis, travel time from home to the cancer center, the time interval from initial symptoms to the start of treatment, treatment-related complications, compliance, and outcome were analyzed. A Cox proportional hazards regression model was applied to investigate the influence of risk factors.

**Results:**

The most common diagnoses in SECI were leukemia (48.8%), lymphomas (24.1%), brain tumors (1%), and other solid tumors (24.7%), compared to 22.3%, 19.3%, 28.6%, and 26.5% in UKK, respectively.

Patients from SECI were younger (5.2 vs. 9.0 years, *P* < 0.001), needed longer travel time to reach the treatment center (1.44 ± 0.07 vs. 0.53 ± 0.03 h, *P* < 0.001), received therapy earlier (7.53 ± 0.59 vs. 12.09 ± 1.01 days, *P* = 0.034), showed less compliance (85.1% vs. 97.1%, *P* < 0.001), and relapsed earlier (7 vs. 12 months, *P* = 0.008). Deaths in SECI were more frequent (47.4% vs. 18.1%) and caused mainly by infection (60% in SECI, 7% in UKK), while in UKK, they were primarily disease-related (79% in UKK, 27.7% in SECI). Differences in overall and event-free survival were observed for leukemias but not for non-Hodgkin lymphoma.

**Conclusions:**

Outcome differences were associated with different causes of death and other less prominent factors.

**Supplementary Information:**

The online version contains supplementary material available at 10.1186/s12887-023-04214-8.

## Background

Substantial progress has been achieved in managing childhood cancers, with overall cure rates reaching 90% in many high-income countries (HICs) [1]. In contrast, the 5-year overall survival (OS) rates in the lower-middle-income countries (LMICs) range from 5 to 60% [1, 2]. More than 80% of children with cancer reside in LMICs [3].

Several factors may contribute to the significant differences in survival rates between children with cancer in HICs and LMICs [4]. A previous descriptive study indicated that survival rates were directly associated with various socioeconomic and health-related factors, including per capita Gross Domestic Product (GDP), Gross National Income, and the ratio of physicians and nurses to the population. The availability of annual governmental funding for health care per capita emerged as a particularly crucial factor [2]. Although all the facts mentioned above, only 1% of child deaths in LMICs are due to cancer, compared to 5% in HICs.

It seems logical for LMICs governments to prioritize their resources towards managing their more prevalent health issues. For instance, children's deaths in LMICs are predominantly caused by diseases such as parasitic and other types of infections and maternal and child health issues related to complications during pregnancy and delivery. Therefore, most of the resources in these countries are directed to solving these problems compared to less frequent diseases such as cancer [5].

A problem that affects childhood cancer epidemiological studies in LMICs is underdiagnosis and under-reporting [6]. Previous studies have shown a higher incidence of childhood cancers in capital cities of LMICs compared to smaller cities, such as Jordan and Honduras. This suggests that children with malignant tumors living in rural areas or distant small cities may have less access to accurate diagnosis and treatment [7, 8]. There is a scarcity of specialized tertiary healthcare centers, particularly in rural areas, resulting in parents traveling long distances with their sick children [9], incurring transportation costs. This prolonged absence from home affects the rest of the family, including loss of income when parents cannot work [10]. The number of pediatric oncologists is limited [6], with a patient-to-specialist ratio ranging from 1:50 to 1:750 in many LMICs, which is inadequate for properly managing such a complex disease [2]. Both quantitative and qualitative shortages of nurses exist, with reports from Uganda indicating a nurse-to-patient ratio of 1:15 during the day and 1:45 at night [6]. A deficiency of well-trained pathologists [6, 10] often results in significant diagnosis delays. In Tanzania, for instance, final pathology reports could take up to one month [2]. LMICs also face a scarcity of both laboratory and radiological diagnostic equipment. Modern diagnostic methods are typically only available in major cities in many LMICs, such as Egypt, Honduras, Morocco, and the Philippines, depriving many patients in rural areas of easy access [2].

In many low-income countries, health insurance does not cover cancer therapy. There is also a shortage of effective drugs, including chemotherapy, antimicrobials, and supportive care drugs. A large study involving 10 LMICs (Bangladesh, Egypt, Honduras, Morocco, the Philippines, Senegal, Tanzania, Ukraine, Venezuela, and Vietnam) found that only three of them (Egypt, Ukraine, and Venezuela) provided childhood cancer patients with access to chemotherapy, antimicrobials, blood products, and radiotherapy [2]. Shortages in other therapy disciplines, such as surgical and radiation oncology, are also observed [6, 11, 12]. The insufficient availability of blood products and lack of screening for infectious diseases further contribute to the challenges [11].

Illiteracy, lack of health-related knowledge, and delays in seeking medical advice are common among parents in LMICs [13]. Treatment abandonment is highly prevalent in these countries, ranging from 25 to 50%, and poses a significant barrier to successful therapy [4, 14, 15].

Research in the pediatric oncology field in different areas of the world can identify the problems in the respective settings, which may lead to finding solutions to overcome the difficulties, thus increasing survival. Adapted protocols to decrease toxicity may be important in resource-limited settings [16].

Approximately 1.800 children are diagnosed with cancer each year in Germany. It has been estimated that the likelihood of a newborn child developing a malignant disease within the first 15 years of life is 0.2% [17]. Unfortunately, there is a lack of epidemiological data on childhood cancer that covers the entirety of Egypt.

Currently, only two cancer Institutes in Egypt are affiliated with universities. The first is the National Cancer Institute, affiliated with Cairo University in Cairo. And the second is the South Egypt Cancer Institute (SECI), affiliated with Assiut University in the southern region of Egypt. As a result, SECI serves as the primary referral center for the entire south part of Egypt. In contrast, as of August 2012, Germany had established 56 pediatric oncology departments [18].

Many previous studies have focused on the characteristics and survival outcomes of children with cancer in HICs, while others have highlighted the challenges faced in managing childhood cancer in LMICs. However, to our knowledge, no study has directly compared these two settings. Therefore, the objective of this study was to conduct a direct comparison between a center in an LMIC (SECI, Assiut University, Egypt) and another in a HIC (Cologne University Hospital, Uniklinik Köln (UKK), Germany) by examining the characteristics and outcomes of children with cancer. This study was carried out by the same researcher who worked in both centers. We aimed to identify the primary factors contributing to the disparity in survival rates for childhood cancers between these two centers. By identifying these differences, we hope to identify transferable structures and conditions that can be implemented in LMICs settings, ultimately improving both the quantity and quality of survival outcomes for children with cancer.

## Methods

This retrospective study analyzed newly diagnosed pediatric patients up to 18 years old with malignant diseases admitted to the pediatric oncology departments at SECI, Assiut University, Egypt, or UKK, Cologne, Germany, between January 1, 2006, and December 31, 2010. We excluded patients with relapse at the time of the first presentation, those who received prior chemotherapy outside SECI or UKK, patients referred to other hospitals or who abandoned therapy before or after diagnosis or initial surgery and before starting chemotherapy, undiagnosed patients, and patients with no available data. Pediatric patients admitted to other departments at SECI (*n* = 235) or who died shortly after admission and before reaching a final diagnosis were also excluded.

The available data from electronic or paper-based reports of all included patients were retrospectively reviewed to gather information on various aspects. This included the duration between the patient's initial symptoms and their first presentation at the cancer center (in weeks), the date of the first presentation at the pediatric oncology department, age, gender, diagnosis, and disease stage at presentation. Additionally, data on travel time from home to the cancer center (in hours, estimated using Google Maps), the duration from first presentation to the start of therapy, treatment-related severe adverse events (graded as 3 or 4 according to the common terminology criteria for adverse events, CTCAE version 4.03) [19], compliance with therapy, site, and date of disease progression or relapse if occurred, date of the last visit, prior clinical status, and causes and date of death if applicable. In cases with combined causes of death (therapy-related and tumor progression), the predominant cause of death was determined by two expert physicians or attributed to the unknown category.

Compliance in this study referred to the acceptance of the proposed therapy and adherence to the prescribed protocols. Treatment refusal was defined as the act of rejecting any treatment for a potentially curable malignant disease after a final diagnosis had been made. Non-compliance with therapeutic rules encompassed situations such as unexplainable delays in scheduled treatment appointments for one or more consecutive weeks during ongoing therapy, refusal of further therapy or recommended investigations, or abandonment of treatment. Abandonment of therapy was determined when patients missed four or more consecutive weeks of treatment without returning to continue their therapy.

### Statistics

The "Statistical Package for Social Science" (SPSS) version 22 was used for the statistical analysis. Descriptive statistics such as number, percentage, mean, median, and standard error were calculated. Group comparisons between categorical data were performed using the chi-square test, while the Mann–Whitney-U test was used for continuous non-parametric data. Survival analysis was conducted using the Kaplan–Meier method, measuring overall survival from the day of the first presentation until an event (death of any reason) or of the last known information if no event occurred. Event-free survival (EFS) was measured from the day of the first presentation until an event (tumor relapse or progression or death of any reason) or the last information if no event occurred.

Survival curves between groups were compared using the log-rank test. A Cox proportional hazards regression model was applied to analyze factors influencing survival. Factors with clinical value, identified through univariate analysis, such as age, gender (male vs. female), duration between initial symptoms and first presentation at the cancer center, travel time from home to the cancer center, duration from first presentation to the start of therapy, and treatment-related severe adverse events (yes vs. no), were included as covariates in the model. A backward selection approach was used with a likelihood ratio test p-value for the threshold of < 0.05 for inclusion.

Data have been collected from 1/7/2011 till 30/11/2011 in both countries. The data lock was set on 30/9/2011 in the SECI group and 30/11/2011 in the UKK group. Ethical approval was obtained from the local ethical committee in SECI and the Institutional Review Board in Cologne University.

## Results

A Comparison of the basic structure of the pediatric oncology department in SECI and UKK is presented in Supplemental Table S1. For detailed information regarding the included and excluded patients in both institutions, please refer to Supplemental Figs. 1 and 2.

### Patients' characteristics of included cohort

A total of 740 patients were included in the study. Among them, 502 patients (291 males and 211 females, ratio 1.4:1) initiated therapy at the Pediatric Oncology Department, SECI, while 238 patients (139 males and 99 females, ratio 1.4:1) started treatment at the Pediatric Oncology Department, UKK.

The patients from SECI were younger than those from UKK, with mean ages of 5.2 and 9.0 years, respectively (*P* < 0.001). The distribution of diagnoses also varied between the two centers. The most common diagnoses in SECI were leukemia (48.8%), lymphomas (24.1%), brain tumors (1%), and other solid tumors (24.7%), compared to 22.3%, 19.3%, 28.6%, and 26.5% in UKK, respectively (Table 1). There were differences in the frequency of oncological risk groups within specific diagnoses. High-risk acute lymphoblastic leukemia (ALL), according to the Berlin-Frankfurt-Münster (BFM) protocol, was more prevalent in SECI (61.8% vs. 24.3% in UKK, *p* < 0.001), as well as stage III and IV of Non-Hodgkin lymphoma (NHL) (86.3% vs. 62.5%, *P* = 0.040). In patients with neuroblastoma, stage 4 was significantly more frequent in SECI than UKK (90.2% vs. 42.9%, *p* = 0.001). No significant differences were observed between the two groups regarding advanced and non-advanced Wilms tumor and Hodgkin lymphoma stages.

**Table 1:** Comparison of pediatric cancer diagnosis and age of patients between SECI and UKK

Diagnosis	SECI (*N* = 502)			UKK (*N* = 238)			*p*-value for the difference in diagnosis	*p*-value for the difference in age
	Number	Mean	SEM	SEM	Mean	SEM		
		Age (years)			Age (years)			
ALL	186	6.80	0.31	37	7.77	0.87	< 0.001	0.486
AML	58	8.46	0.61	13	7.43	1.58	0.009	0.429
CML	1	0.58		3	11.36	5.67	0.193	0.655
Brain tumors	5	6.70	2.82	68	8.58	0.54	< 0.001	0.450
NHL	80	7.12	0.43	16	11.88	1.00	0.056	< 0.001
Hodgkin lymphoma	41	9.39	0.58	30	14.00	0.64	0.056	< 0.001
Neuroblastoma	41	3.09	0.51	14	2.63	0.50	0.268	1.000
Wilms tumor	29	3.08	0.47	7	2.48	0.70	0.094	0.644
Bone tumors	9	11.33	1.13	15	11.71	1.12	0.003	0.528
Germ cell tumors	9	4.20	1.06	8	10.84	2.47	0.183	0.111
Rhabdomyosarcoma	11	3.65	0.83	6	8.30	2.97	0.780	0.227
Hepatoblastoma	6	2.68	0.92	4	5.29	2.75	0.847	0.831
Retinoblastoma	4	2.95	0.46	0			0.399	
PNET	6	8.23	2.02	0			0.210	
Other sarcomas	3	9.20	3.84	7	5.65	2.10	0.025	0.253
Other carcinomas	6	13.17	0.48	2	11.50	1.50	0.956	0.302
LCH	3	3.47	1.25	8	6.32	2.04	0.010	0.410
MDS	4	7.00	3.08	0			0.399	

*SECI* Indicates South Egypt Cancer Institute, *UKK* Cologne University Hospital, *SEM* Standard error of the mean, *ALL* Acute lymphoblastic leukemia, *AML* Acute myeloid leukemia, *CML* Chronic myeloid leukemia, *NHL* Non-Hodgkin lymphoma, *PNET* Primitive neuroectodermal tumor, *LCH* Langerhans cell histiocytosis and *MDS* Myelodysplastic syndrome

### Time factor characteristics 

The travel time (hours) from the patient's home to the cancer center was significantly longer for patients in SECI compared to those in UKK in most disease categories (1.44 ± 0.07 vs. 0.53 ± 0.03 h, respectively, *P* < 0.001).

The time interval between the first symptom and the first presentation at the cancer center (weeks) did not show a significant difference between the two groups overall. However, subgroup analysis revealed that this time interval was notably longer in patients with ALL and patients with Wilms tumor in SECI. There was no gender difference in both groups regarding the median time till presentation (4 weeks).

The time interval between the first presentation at the cancer center and the start of treatment was significantly shorter in SECI overall. Subgroup analysis demonstrated a statistically significant shorter time till starting treatment in bone tumors, Wilms tumors, and other carcinomas for SECI compared to UKK. The time till the start of therapy in patients with ALL was significantly longer in SECI (Table 2).

**Table 2:** Duration of travel between patients' home and the treating center, time from the first symptom till the first visit to the cancer center, and time from the first visit to the center till the start of treatment in children with cancers between SECI and UKK

	Duration of travel (hours) between patients' homes and the treating center					Time interval (weeks) between the first symptom and first visit to the cancer center					Time interval (days) between the first visit to the cancer center and the start of treatment*				
	SECI		UKK		*p*-value	SECI		UKK		*p*-value	SECI		UKK		*p*-value
Final Diagnosis	Mean	SEM	Mean	SEM		Mean	SEM	Mean	SEM		Mean	SEM	Mean	SEM	
ALL	1.46	0.11	0.39	0.05	< 0.001	5.28	0.52	3.13	0.60	0.005	4.71	0.28	1.97	0.29	< 0.001
AML	1.31	0.17	0.39**	0.08	0.002	4.89	0.85	7.15	3.19	0.422	4.93	0.39	6.08	1.43	0.654
CML	2.60		0.53	0.19	0.180	3.00		16.52***	15.74	0.655	17.00		9.33	5.46	0.655
Brain tumors	1.70	0.64	0.69**	0.10	0.074	10.60	5.64	9.92	1.56	0.750	16.25	9.66	12.48	2.28	0.153
NHL	1.31	0.16	0.37	0.05	< 0.001	6.02	0.63	7.88	3.15	0.344	9.01	1.39	10.13	2.30	0.307
HL	1.32	0.23	0.48**	0.05	0.079	16.50	3.46	10.75	2.38	0.217	13.12	2.56	15.60	2.14	0.091
Neuroblastoma	1.45	0.18	0.58	0.09	0.009	6.27	1.26	6.92	2.46	0.250	7.05	1.46	17.93	8.30	0.130
Wilms tumor	1.62	0.29	0.48^&^	0.10	0.083	3.17	0.71	1.18	0.69	0.020	4.93	0.95	15.43	5.88	0.049
Bone tumors	2.02	0.60	0.62	0.11	0.257	16.16	5.40	8.93	0.85	0.853	9.78	3.70	16.00	2.02	0.037
GCT	0.98	0.37	0.48	0.05	0.923	19.00	10.25	4.89	1.51	0.265	27.89	22.80	15.00	6.07	0.885
RMS	2.07	0.58	0.65	0.16	0.096	11.18	3.75	4.50	1.20	0.386	6.20	1.40	14.50	4.12	0.070
Hepatoblastoma	0.98	0.46	0.25	0.08	0.087	4.67	1.50	5.60	3.49	0.516	11.50	2.81	12.00	3.56	0.831
Retinoblastoma	1.13	0.69				35.00	20.62				19.00	13.93			
PNET	1.31	0.36				4.07	0.89				14.83	4.23			
Other sarcomas	2.49	1.02	0.48	0.11	0.053	5.67	1.45	4.45	2.05	0.491	8.00	2.00	21.71	7.41	0.462
Other carcinomas	1.60	0.66	0.62	0.02	0.505	8.07	3.56	13.00	1.00	0.241	10.17	2.61	26.50	3.50	0.044
LCH	2.23	0.97	0.37	0.05	0.219	6.00	1.15	6.33	2.72	0.469	22.00	19.00	20.38	3.06	1.000
MDS	1.92	0.58				10.48	6.40				18.00				
Total	1.44	0.07	0.53^#^	0.03	< 0.001^@^	7.14	0.51	7.63	0.68	0.716^@^	7.53	0.59	12.09	1.01	0.034^@^

*SECI* Indicates South Egypt Cancer Institute, *UKK* Cologne University Hospital, *SEM* Standard error of the mean, *ALL* Acute lymphoblastic leukemia, *AML* Acute myeloid leukemia, *CML* chronic myeloid leukemia, *NHL* Non-Hodgkin lymphoma, *HL* Hodgkin lymphoma, *GCT* Germ cell tumors, *RMS* Rhabdomyosarcoma, *PNET* Primitive neuroectodermal tumor, *LCH* Langerhans cell histiocytosis and MDS, Myelodysplastic syndrome

^*^In the SECI group, the following cases have been excluded from the calculations of time till the start of treatment: 5 cases ALL, 3 AML, 1 LCH, 3 MDS, 3 NHL, one other sarcoma, one case brain tumor, and one RMS, since they received just supportive therapy and died early. In UKK, one patient with a brain tumor and another with osteosarcoma after renal transplantation were excluded for the same reason

^**^Exclusion of 1 case coming from abroad in UKK

^***^Median time till the presentation of CML in UKK was seven days

^&^ Exclusion of 2 cases coming from abroad in UKK

^#^ Exclusion of 5 cases in UKK coming from abroad

^@^ Independent T-test for mean

### Therapy-related complications

The two groups had no significant difference in the overall incidence of complications. The percentage was slightly higher in UKK compared to SECI, with rates of 66.4% vs. 62.2%, respectively (Supplemental Table S2). However, notable differences were observed in the types of complications. The UKK group had a higher occurrence of febrile neutropenic infections, fungal infections, peripheral neuropathies (including constipation), central nervous system complications, bone and joint affections, endocrine abnormalities, auditory impairment, cardiomyopathy, and postoperative complications. On the other hand, the SECI group had a higher occurrence of hepatopathies, infections with hepatitis B/C, and widespread viral infections.

### Compliance in both centers

Patients in UKK exhibited significantly higher compliance with therapy than SECI, with rates of 97.1% and 85.1%, respectively (*p* < 0.001).

### Relapse rate

There was no statistical difference in the overall incidence of relapse between the two groups, with rates of 13.9% and 15.5%, respectively. However, the SECI group experienced relapses at an earlier time compared to the UKK group, with a median time from diagnosis to relapse of 7 (1–43) months and 12 (0.75–37) months, respectively (*p* = 0.008).

### Clinical outcomes

**Fig. 1:**
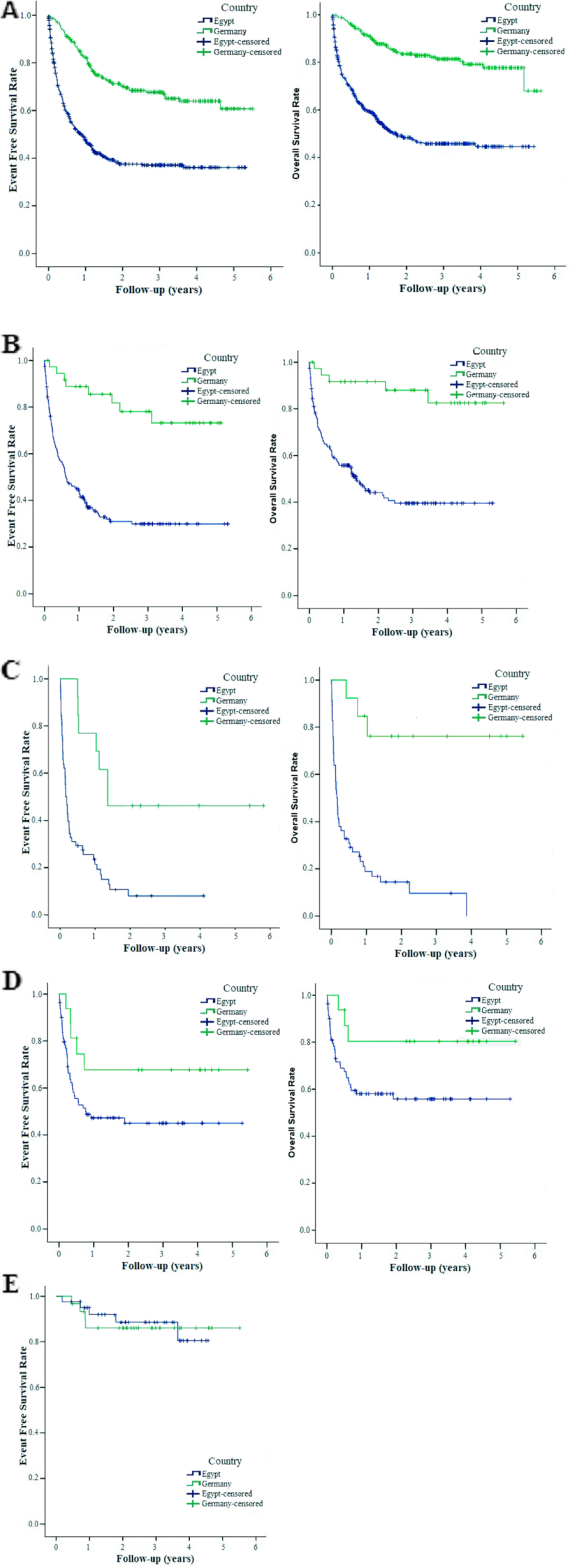
Overall event-free survival and overall survival for children with cancer at South Egypt Cancer Institute (SECI) and Cologne University Hospital (UKK). **A** Whole groups. SECI (*N* = 502), UKK (*N* = 238), 5-years EFS ± SEM 0.36 ± 0.03 vs. 0.61 ± 0.05, in SECI and UKK groups, respectively (*p* < 0.001). 5-years OS ± SEM 0.45 ± 0.03 vs. 0.78 ± 0.03, in SECI and UKK groups, respectively (*p* < 0.001). **B** Children with acute lymphoblastic leukemia. SECI (*N* = 186), UKK (*N* = 37), 5-years EFS ± SEM 0.30 ± 0.04 vs. 0.73 ± 0.08, in SECI and UKK groups, respectively (*p* < 0.001). 5-years OS ± SEM 0.40 ± 0.04 vs. 0.83 ± 0.08, in SECI and UKK groups, respectively (*p* < 0.001). **C** Children with acute Myeloid leukemia. SECI (*N* = 58), UKK (*N* = 13), 5-years EFS ± SEM 0.08 ± 0.04 vs. 0.46 ± 0.14, in SECI and UKK groups, respectively (*p* < 0.001). 5-years OS ± SEM 0.0 ± 0.00 vs. 0.76 ± 0.12, in SECI and UKK groups, respectively (*p* < 0.001). **D** Children with non-Hodgkin lymphoma. SECI (*N* = 80), UKK (*N* = 16), 5-years EFS ± SEM 0.45 ± 0.06 vs. 0.68 ± 0.12, in SECI and UKK groups, respectively (*p* = 0.089). 5-years OS ± SEM 0.56 ± 0.06 vs. 0.81 ± 0.10, in SECI and UKK groups, respectively (*p* = 0.070). **E** Children with Hodgkin lymphoma. SECI (*N* = 41), UKK (*N* = 30), 5-years EFS ± SEM 0.81 ± 0.09 vs. 0.86 ± 0.07, in SECI and UKK groups, respectively (*p* = 0.910). Log-rank test was calculated to compare survival curves between groups

The overall mortality rate was higher in SECI compared to UKK. In the UKK group, 43 patients (18.1%) died, while in the SECI group, 238 patients (47.4%) succumbed to their condition (*p* < 0.001) (Table 3 and Fig. 1).

**Table 3:** Comparison of mortality rate among different pediatric cancers diagnoses between SECI and UKK

Diagnosis	SECI		UKK		*p*-value^§^
	N^*^	%	N^*^	%	
ALL	99/186	53.2%	5/37	13.5%	< 0.001
AML	50/58	86.2%	3/13	23.1%	< 0.001
CML	1/1	100.0%	1/3	33.3%	1.000
Brain Tumors	3/5	60.0%	14/68	20.6%	0.143
NHL	33/80	55%	3/16	18.8%	0.157
Hodgkin lymphoma	0/41	0.0%	0/30	0.0%	
Neuroblastoma	22/41	53.7%	5/14	35.7%	0.395
Wilms Tumor	6/29	20.7%	0/7	0.0%	0.451
Osteosarcoma	4/6	66.7%	3/8	37.5%	0.180
Ewing sarcoma	1/3	33.3%	0/7	0%	
Germ Cell Tumors	1/9	11.1%	0/8	0.0%	1.000
Rhabdomyosarcoma	5/11	45.5%	3/6	50.0%	1.000
Hepatoblastoma	3/6	50.0%	1/4	25.0%	0.895
Retinoblastoma	0/4	0.0%			
PNET	4/6	66.7%			
Other sarcomas	2/3	66.7%	4/7	57.1%	1.000
Other carcinomas	2/6	33.3%	1/2	50.0%	1.000
LCH	1/3	33.3%	0/8	0.0%	0.592
MDS	1/4	25.0%			
Total	238/502	47.4%	43/238	18.1%	< 0.001

*SECI* Indicates South Egypt Cancer Institute, *UKK* Cologne University Hospital, *ALL* Acute lymphoblastic leukemia, *AML* Acute myeloid leukemia, *CML* Chronic myeloid leukemia, *NHL* Non-Hodgkin lymphoma, *PNET* Primitive neuro-ectodermal tumor, *LCH* Langerhans cell histiocytosis and *MDS* Myelodysplastic syndrome

^§^Group comparisons between categorical data were performed using a chi-square test

^*^The number of deaths/number of patients per diagnosis

The OS and EFS outcomes in the most common diseases differed between the two groups. Figure 1 illustrates the superior Kaplan–Meier curves of the UKK group compared to the SECI group in terms of overall EFS and OS (Fig. 1A). This trend was also observed in patients with ALL (Fig. 1B) and acute myeloid leukemia (AML) (Fig. 1C). There was a positive trend for patients with Non-Hodgkin lymphoma (Fig. 1D). Still, no advantage was observed for patients with Hodgkin lymphoma (Fig. 1E). It should be noted that the SECI group had a higher proportion of patients with high-risk ALL compared to the UKK group. However, some diagnoses had too few deaths to allow for a statistically valid comparison. Table 4 presents the differences in mortality causes between the two groups. In the SECI group, most patient deaths (66%) were attributed to therapy-related complications, particularly infections. In contrast, in the UKK group, a larger percentage (79%) of mortalities were due to tumor progression.

**Table 4:** Overall causes of death among pediatric cancer patients between SECI and UKK*

Cause of death	SECI Number of patients that died = 238	UKK Number of patients that died = 43	*p*-Value
1-Related to tumor relapse/ progression	66 (27.7%)	34 (79.1%)	< 0.001
2-Related to therapy (combined causes are possible, e.g., infection and hemorrhage)	157 (66%)	7 (16.3%)	< 0.001
Infection	Total 143 (60%) Septicemia with Fever neutropenia: 91 pneumonia 39 Fungal chest infection 1 Acute gastroenteritis 3 Peritonitis 3 Typhlitis 1 Meningitis 3 Postoperative septicemia due to burst abdomen and intestinal fistula 2	Total 3 (7%) Fungal 2 Viral H1N1 1	< 0.001
Hemorrhage	Total 17 (7.1%) Intracranial 6 Pulmonary 7 Hematemesis 2 External hemorrhage 2	Total 1 (2.3%) Intracranial 1	0.326
Tumor lysis syndrome	13 (5.5%)	0 (0%)	0.230
Chemotherapy toxicity	Total 10 (4.2%) Methotrexate 3 Others 7	Total 5 (11.6%) Methotrexate 1 Others 1 BMT 2 CD 33 antibodies cytokine syndrome 1	0.104
Anemic heart failure	9 (3.8%)	0 (0%)	0.363
Acute Renal Failure	2 (0.8%)	0 (0%)	1.000
2^nd^ Malignancy	0 (0%)	1 (2.3%)	0.153
3-Unknown cause	15 (6.3%)**	2 (4.6%)	0.944

*SECI* Indicates South Egypt Cancer Institute, *UKK* Cologne University Hospital and *BMT* Bone marrow transplantation

*Many patients had more than one cause of therapy-related mortality

** one patient died at home and another on his way before reaching SECI

### Comparison of risk factors affecting survival

The univariate analysis of mortality risk factors revealed different results in both groups. In the SECI group, higher mortality was associated with a longer time from initial symptoms to a presentation at the cancer center, non-compliance with therapy, and complications or relapse. On the other hand, in the UKK group, higher mortality was associated with patients living far away from the treating center and complications or relapse.

The multivariate analysis further confirmed these findings. In the SECI group, patients had an increased risk of death if they experienced complications or had a long time from initial symptoms to presentation at the cancer center. In the UKK group, patients living far away from the cancer center and those who suffered complications were at a higher risk of death (Table 5).

**Table 5:** Cox regression analysis for risk factors affecting overall survival rate (multivariate analysis) in SECI and UKK groups

		B	Significance	Exp (B)	95.0% CI	
					Lower	Upper
SECI	The distance of the cancer center from home		0.550			
	Time from symptoms till the first presentation	0.017	0.026	0.983	0.966	1.000
	Treatment-related severe adverse events (yes vs. no)	0.580	< 0.001	1.786	1.315	2.425
	Time from the first admission till the start of treatment		0.277			
UKK	Distance of the cancer center from home	0.553	0.015	1.739	1.207	2.504
	Time from symptoms till the first presentation		0.535			
	Treatment-related severe adverse events (yes vs. no)	0.945	0.014	2.573	1.124	5.888
	Time from the first admission till the start of treatment		0.765			

*SECI* Indicates South Egypt Cancer Institute and *UKK* Cologne University Hospital

## Discussion

In this retrospective study, the management of childhood cancers was compared between two centers: one located in an LMIC (Egypt) and the other in a HIC (Germany). The SECI included a significantly larger number of patients than the UKK group. The common diagnoses in SECI were leukemia, lymphomas, and solid tumors, however brain tumors were rarely observed. On the other hand, brain tumors were common in UKK.

Compared to the patients in UKK, those in the SECI group were younger, had longer travel times to reach the treatment center, received therapy earlier because of substantial outside initial diagnosis, exhibited less treatment compliance, and experienced earlier relapses. The SECI group had a higher frequency of deaths, primarily attributed to delayed presentation and complications, predominantly caused by infections. In contrast, the deaths in the UKK group were mainly related to the disease itself.

It is well acknowledged that this study is not a formal epidemiological study that encompasses the entire population of children in a specific area. Nonetheless, the study was over five years, providing a real-world perspective without any exceptions. The research reflects the daily work carried out by the same researcher in both a HIC and an LMIC. It compared the real everyday problems obstructing proper management of childhood cancers in a center in an LMIC through a comparison with another center in a HIC.

The significant disparity in survival rates between UKK and SECI appeared to be linked to several unique problems encountered in tumor management in SECI. These challenges include a limited number of specialized treatment centers in Egypt, which results in overcrowded patient rooms where multiple patients often stay together, a higher patient-to-healthcare team ratio, and long distances between patients' homes and the cancer center. Furthermore, there are deficiencies in certain diagnostic facilities and intermittent shortages of blood products and drugs. These factors contribute to misdiagnoses, late presentation of patients at advanced stages, and non-compliance with therapy.

This study has limitations due to the small sample sizes and the random selection of centers for analyses. However, the comparison made in this study offers valuable insights and identifies areas where immediate actions can be taken. Conducting larger-scale research nationally or including more hospitals would be beneficial, particularly when a comprehensive cancer registry representing a whole country in an LMIC is available.

The findings of this study shed light on the challenges faced by SECI, which are likely applicable to many similar centers in LMICs. One major issue is the deficiency of well-established pediatric oncology centers that cover all regions of Egypt, as better healthcare facilities are predominantly available in the capital and major cities [20]. As a result, SECI receives a higher number of patients compared to UKK. It should be noted that many children come to SECI for RTH or surgery without being referred to the Pediatric Oncology Department, highlighting the shortage of these specialties in most parts of southern Egypt. The main points related to this problem are as follows:

A A significant difference is observed in the ratio of new patients to physicians, with a ratio of 12:1 in SECI compared to 7:1 in UKK. The low physician-to-patient ratio is not solely due to a lack of available hospital positions. Still, it is also a result of the wish of many pediatricians in smaller cities in Egypt who are not inclined to specialize in oncology due to the lack of job opportunities in their respective areas.
B There was a significant burden on the nursing staff at SECI. The ratio of nurses to patients beds in SECI was 13:54, whereas, in UKK, the ratio was 25:21.
C The patients' rooms in SECI were overcrowded, each accommodating eight beds. Each bed was occupied by the patient, their mother, and potentially their siblings. This overcrowding contributes to a high rate of infection. The absence of guest houses for the parents further exacerbates this issue.
D Parents faced the challenge of long-distance travel with their sick children, particularly for patients in SECI, and they had to bear the transportation costs. This problem led some parents to prefer referrals to smaller centers with fewer facilities but closer proximity to their homes (*n* = 39 patients). Tragically, in some cases (*n* = 2), patients who had initiated therapy in SECI and lived far away passed away either at home or during the journey before reaching SECI. Furthermore, 21 patients presented to SECI in critical condition and succumbed shortly after arrival, within minutes to hours, before a final diagnosis or anticancer therapy could be initiated. Many of these patients experienced significant delays in receiving an accurate diagnosis due to misdiagnosis by inexperienced healthcare providers in their rural areas.

Compounding this problem, a number of non-malignant cases were misdiagnosed as malignant tumors by inexperienced healthcare providers in rural areas and were subsequently referred to SECI. These misdiagnosed patients accounted for 14% of all patients seen at the pediatric oncology department in SECI, further straining the financial resources of the center as they also required supportive care until an accurate diagnosis was established. This issue could be alleviated by improving transportation infrastructure, establishing a dedicated parents' house at SECI, and establishing well-equipped primary or secondary cancer care centers that operate under the supervision of the tertiary cancer care center and employ well-trained personnel.

Unfortunately, Egypt lacks a childhood cancer registry that could be used for comparison with SECI's rates of cancer types and survival rates. In most LMICs, registries are often inaccurate and limited to local hospitals [21, 22]. Accurate hospital-based registries are crucial in countries without a national cancer reistry to provide insights into patients' demographic and clinical characteristics, treatment outcomes, and challenges, ultimately leading to national solutions. It is essential to review these data periodically [23].

Among the patients in SECI, the most common tumors were leukemia, followed by NHL, Hodgkin lymphoma, neuroblastoma, and Wilms tumor. The high proportion of leukemia cases (approximately 50% of all SECI patients) is likely due to the relatively better supportive care available at SECI compared to the other centers in the south of Egypt, which used to refer leukemia patients to SECI. In contrast, brain tumors were infrequent as other cancer centers in south Egypt would directly refer brain tumor patients to Cairo, where superior neurosurgical care facilities were accessible. It is worth noting that UKK is renowned for its expertise in managing brain tumors, thus attracting patients from more distant areas than other patients in Cologne. Brain tumors were the most prevalent tumor type among this group.

In the SECI group, the time from diagnosis to the initiation of treatment was significantly shorter overall, especially in patients with bone tumors and Wilms tumors. This was because many of these patients had previously been admitted to the university hospital and were referred to SECI with all investigations completed and ready to start chemotherapy. However, in patients with ALL, the time until treatment initiation was significantly longer in SECI. This was because bone marrow aspirates and biopsies were only performed once or twice a week at SECI, and these procedures were carried out exclusively by clinical pathologists within the center. In contrast, at UKK, pediatric oncologists performed these procedures daily and personally reviewed the bone marrow slides.

Although both centers used the same therapy protocols, UKK had a slightly higher incidence of complications. The variation in complication rates may be misleading because UKK had a more comprehensive system for documenting complications, including printed documentation of severity grades and a well-defined follow-up program for cancer survivors. In contrast, due to financial constraints at SECI, investigations were primarily conducted based on clinical suspicion of organ function deterioration.

Analysis of therapy-related complications revealed several problems that SECI encountered:

1. The deficiency of blood products resulted in anemic heart failure (*n* = 9) and death from hemorrhage (*n* = 17).
2. There was a high incidence of tumor lysis syndrome (3%) in SECI, while no patient developed this syndrome in UKK. This can be attributed to the higher proportion of patients with ALL and NHL compared to UKK. It can also be attributed to the higher number of patients presenting with advanced stages of the disease and a large tumor burden in SECI.
3. A high prevalence of hepatitis B virus (HBV) and hepatitis C virus (HCV) infection (*n* = 58) was observed in SECI. The prevalence of HCV (8%) was lower than that reported in a previous study conducted on 35 pediatric ALL survivors in another center in Egypt (28.6%) [24]. It is worth mentioning that the serological status of patients regarding HBV and HCV at the time of their first presentation to SECI and at the end of therapy was not routinely determined during the study. Serology tests were only conducted if the patient developed jaundice or had elevated liver enzyme levels.
4. High rates of infection were observed in the SECI group. It is important to highlight that 143 patients (28.5% of the cohort in SECI) died due to infection. Infections pose a significant risk to the health and survival of pediatric cancer patients, and these findings underscore the need for comprehensive infection prevention and control measures in managing childhood cancer.

A large-scale study involving 101 countries worldwide, using survey and population data, revealed that out of 155,088 children under 15, 23,854 (15%) were newly diagnosed with cancer but abandoned therapy. Alarmingly, 83% of these new childhood cancer cases and 99% of treatment abandonment were reported in LMICs [25]. Limited data are available from Arab countries, especially regarding therapy abandonment [20]. The findings regarding non-compliance with therapy in SECI in this study were consistent with results from other LMICs studies [26]. UKK demonstrated significantly higher compliance with treatment than the SECI group, with rates of 97.1% vs. 85.1%, respectively. No patient in UKK abandoned treatment or was discharged in demand. It is important to note that SECI provides free-of-cost cancer treatment, including hospital care, medical supplies, and investigations to most patients.

Although the relapse rate did not differ between SECI and UKK, the SECI group experienced earlier relapses. However, this finding must be interpreted regarding differences in diagnoses, risk groups, presentation age, and other factors. Notably, patients with AML in SECI had a seemingly lower relapse rate than UKK (6.9% vs. 46.2%, *p* = 0.001). This can be attributed to the high death rate (86.2%) observed among these patients during their initial chemotherapy. It is important to acknowledge that at the time of this study, hematopoietic stem cell transplant services were unavailable throughout the south of Egypt. Patients needing such treatment had to travel to Cairo, which often entailed a long waiting list. In contrast, Germany has multiple centers offering this service, ensuring nationwide accessibility.

This study aimed to identify factors affecting survival in both groups to contribute to global improvements in survival rates. The relapse occurrence at the first presentation was excluded from further analysis, as its worse prognosis is well-known in each disease category. Analyzing the effect of non-compliance with therapy on survival was challenging due to the low numbers of non-compliant cases in UKK and the unknown fate of approximately 50% of non-compliant patients in SECI who either abandoned or refused therapy. A comparison of survival and related factors between both groups within the same diagnosis and stage would be more useful, but this was not one of the aims of this study.

The significant difference in OS and EFS between the two groups is unsurprising. The study highlighted the importance of better management of complications and early presentation of patients at the time of their initial diagnosis to the cancer center, which are crucial factors for improving the OS in SECI. Effective management of complications and increased awareness among the general population and healthcare providers, particularly in rural areas, regarding the signs and symptoms of childhood cancers, can help decrease the time lag until referral to the cancer center and improve survival rates in these children.

Analysis of the causes of death between the groups revealed another crucial finding: Deaths in UKK were primarily attributed to relapse and disease progression (79.1%). In comparison, deaths in SECI were predominantly related to therapy (66%). Therapy-related factors can be considered avoidable causes. These results align with studies conducted in Indonesia by Saskia Mostert [13], which demonstrated that infection and bleeding were the most common causes of treatment-related death. Adapting therapy protocols in LMICs based on the availability and efficacy of supportive care may lead to better chances of survival, as opposed to using the original aggressive protocols used in HICs.

Enhancing the model of care for LMICs requires several crucial elements, including raising public awareness through education, improving the training of healthcare professionals, strengthening cancer services, conducting research that is relevant to local needs, establishing regional hospital networks, fostering international collaboration, and ensuring access to health insurance [10].

In conclusion, there is a significant disparity in managing childhood cancers and subsequent survival rates between HICs and LMICs. The direct institutional comparison of pediatric cancer patients between a HIC and an LMIC can help identify factors associated with poorer outcomes in LMICs. Many specific problems related to tumor management in LMICs were highlighted, such as late presentation, non-compliance with therapy, deficiencies in specialized treatment centers, and shortages of well-trained specialists and nurses in pediatric oncology. Outcome differences were associated with different causes of death and other less prominent factors.

Our recommendations for improving cancer care in SECI, as well as other LMICs, are: Proper management of therapy-related complications, improvement of cancer awareness as a disease can affect children in general and its principal curability, efforts are needed to reduce the abandonment of therapy and establishment of country-based cancer registry.

### Supplementary Information

**Additional file 1: Supplemental Table S1.** Comparison between the Pediatric Oncology Department in South Egypt Cancer Institute (SECI) (Egypt) and in Cologne University Children’s Hospital (UKK) (Germany). **Supplemental Table S2.** Comparison of rate of therapy-related complications among pediatric cancer patients between SECI and UKK*. **Supplemental Fig. 1.** Children patients first presented between 2006 and 2010 to South Egypt Cancer Institute (SECI). **Supplemental Fig. 2.** Children patients first presented between 2006 and 2010 to the pediatric hematology/oncology department in Cologne University Hospital (UKK).

## Data Availability

The datasets generated during and/or analyzed during the current study are available from the corresponding author upon reasonable request.
