# Supplementary material for: Comparing presentations and outcomes of children with cancer: a study between a lower-middle-income country and a high-income country
Source: BMC Pediatr. 2023 Sep 5;23:443. doi: 10.1186/s12887-023-04214-8 (PMC10478379; doi:10.1186/s12887-023-04214-8)
Supplement: Supplementary file 1 — Additional file 1: Supplemental Table S1. Comparison between the Pediatric Oncology Department in South Egypt Cancer Institute (SECI) (Egypt) and in Cologne University Children’s Hospital (UKK) (Germany). Supplemental Table S2. Comparison of rate of therapy-related complications among pediatric cancer patients between SECI and UKK*. Supplemental Fig. 1. Children patients first presented between 2006 and 2010 to South Egypt Cancer Institute (SECI). Supplemental Fig. 2. Children patients first presented between 2006 and 2010 to the pediatric hematology/oncology department in Cologne University Hospital (UKK). [file 12887_2023_4214_MOESM1_ESM.docx]

**SUPPLEMENTAL TABLE S1 Comparison between the Pediatric Oncology Department in South Egypt Cancer Institute (SECI) (Egypt) and in Cologne University Children’s Hospital (UKK) (Germany)**

|  | SECI | UKK |
| --- | --- | --- |
| Specialty of the department | Pediatric oncology | Pediatric hematology oncology |
| Number of beds | 50+4 in intermediate care unit | 15+6 in the day care unit |
| Number of beds per room | 8 | 5 rooms with 2 beds, 5 rooms with 1 bed for the patient and one for the mother. |
| Outpatient clinic | Twice weekly | Daily |
| Number of new patients/ year | 150-200 | 65-80 |
| Number of physicians | 17 | 12 |
| Number of nurses | 13 | 25 |
| Doctor: patient ratio | 1:12 | 1:7 |
| Main source of referral | Assiut, New valley, Red See governorates and whole cities in south of Egypt | Cologne and surrounding area |
| Availability of pediatric intensive care unit | Available | Available |
| Regular access to irradiated blood products | Often, but not always available | Available |
| Other essential coworkers, e.g. dietitian, psychologists, physiotherapists | Absent | Present |

SECI indicates South Egypt Cancer Institute; and UKK, Cologne University Hospital.

**SUPPLEMENTAL TABLE S2 Comparison of rate of therapy-related complications among pediatric cancer patients between SECI and UKK***

| Complication | SECI | UKK | p-value§ |
| --- | --- | --- | --- |
| Chemotherapy-induced peripheral neuropathy/constipation | 5 (1%) | 21 (8.8%) | < 0.001 |
| CNS (total) | 23 (4.6%) | 23 (9.7%) | 0.009 |
| -Toxic encephalopathy / Hysteria | 3 (0.6%) | 13 (5.5%) |  |
| -DCL/convulsions | 8 (1.6%) | 3 (1.3%) |  |
| -Brain infarction | 1 (0.2%) | 3 (1.3%) |  |
| -Meningitis | 3 (0.6%) | 2 (0.8%) |  |
| -Post RTH syndrome | 0 (0%) | 1 (0.4%) |  |
| -Intra cranial hemorrhage | 6 (1.2%) | 1 (0.4%) |  |
| -Sudden bilateral blindness | 1 (0.2%) | 0 (0%) |  |
| -Paraplegia | 1 (0.2%) | 0 (0%) |  |
| Fever neutropenia/ septicemia** | 96 (19.1%) | 74 (31%) | < 0.001 |
| Fungal infection** | Chest 2 (0.4%) | Total 14 (5.9%)  -Systemic aspergillus infection 6  -Chest 5  -Candida 3 including one case with candida peritonitis | < 0.001 |
| Bone and joint | Total 3 (0.6%)  -Septic arthritis 1  -Osteomyelitis 2 | Total 8 (3.4%)  -Osteonecrosis 5  -Osteoporosis and fracture 2  -Kyphosis 1 | 0.007 |
| Endocrine** | Total 1 (0.2%)  -Diabetes Mellitus 1 | Total 24 (10%)  -Hypothyroidism 11  -Diabetes Mellitus 8  -Suprarenal insufficiency 2  -Hashimoto thyroiditis 1  -Hyperparathyroidism 1  -Stunted growth 1 | < 0.001 |
| Chest | Total of 74 cases (14.7%)  -Pneumonia associated with respiratory failure 69, including intrapulmonary hemorrhage 7  -Pleural effusion 2  -Pneumothorax 2  -Interstitial pulmonary fibrosis 1 | Total of 35 cases (14.7%)  -Pneumonia 19  -Respiratory failure 9  -H1N1 infection 1  -Mycoplasma Pneumonia 1  -Pleural effusion 3  -Pneumothorax 1  -Interstitial pulmonary fibrosis 1 | 1 |
| Pancreatitis | 1 (0.2%) | 5 (2.1%) | 0.015 |
| Liver | Total 66 (13.4%)  -Toxic hepatopathy 60  -Hepatic encephalopathy 6 | Total 11 (4.6%)  -Toxic Hepatopathy 6  -Veno-occlusive disease 5 | < 0.001 |
| Renal | Total 24 (4.8%)  -Tumor lysis syndrome 14  -Acute renal failure 8  -Hemorrhagic cystitis 2 | Total 5 (2.1%)  -Acute renal failure 5 | 0.104 |
| Central venous line complications | not used it in SECI | Total 36 in 32 cases (13.4%)  -Infections 13  -Venous thrombosis 10  -Dislocation 8  -Obstruction 3  -Pleural effusion/Pneumothorax 2 |  |
| Peripheral venous line complications | Total 21 (4.2%)  -Local site cellulitis 13  -Hand/ Foot Abscess 8 | Total 2 (0.8%)  -Extravasation 2 | 0.012 |
| Gangrene | Total 6 (1.2%)  -Lower lip 3  -Nasal 2  -Eyelid 1 | 0 (0%) |  |
| Postoperative complications | Total 18 in 14 cases (2.8%)  A-Technical problems:7  -Postoperative intestinal obstruction due to adhesions 3  -Hydropneumothorax after chest tapping 1  -Nerve injury (postoperative stool and urine incontinence) 1  -Convulsions and DCL after bone marrow aspirate (hypoxia) 1  -Postoperative intestinal fistula 1  B-Infections:5  -Wound infection 4  -Graft infection 1  C-Others:6  -Burst abdomen 3  -Incisional Hernia 2  -Toxic ileus/perforation 1 | Total 19 in 18 cases (7.6%)  A-Technical problems:10  -Shunt insufficiency 4  -Shunt wrong way to vagina 1  -Locked-in syndrome/contracture after tumor resection 2  -Chronic subdural hematoma 1  -Cranial nerve injury 2  B-Infections:8  -Ventriculitis/encephalitis after brain surgery 4  -Shunt/ prosthesis infection 3  -Bacteremia after surgery 1  C-Others:1  -Postoperative posterior fossa syndrome 1 | 0.006 |
| Methotrexate-toxicity (high level) | Total 22 (4.4%)  -With jaundice 9  -With renal failure 3  -Without any 10 | Total 11 (4.6%)  -With jaundice 2  -With renal failure 2  -Without any 7 | 0.851 |
| Ear | Total 5 (1%)  -Deafness*** 3  -Acute otitis media 1  -Chronic suppurative otitis media 1 | Total 16 (6.7%)  -Deafness 16 (Including even mild degree deafness) | < 0.001 |
| HBV infection** | Total 19 (3.8%)  -With jaundice 10 | 0 (0%) | 0.001 |
| HCV infection** | Total 33 (6.8%)  -With jaundice 14 | 0 (0%) | < 0.001 |
| Combined HCV and HBV infection** | Total 6 (1.2%)  -With jaundice 4 | 0 (0%) | 0.185 |
| Blood | Total 10 (2%)  -Anemic heart failure 9  -Deep venous thrombosis 1 | Total 1 (0.4%)  -Iron overload from repeated blood transfusion 1 | 0.116 |
| Heart** | Total 4 (0.8%)  -Cardiomyopathy 4 | Total 7 (2.9%)  -Cardiomyopathy 6  -Prolonged QT interval 1 | 0.044 |
| Gastro-intestinal tract | Total 15 (3%)  -Typhlitis/ Peritonitis 5  -Severe acute gastroenteritis with dehydration 4  -Hematemesis 3  -Intussusception/intestinal obstruction 2  -Severe perianal infection 1 | Total 3 (1.3%)  -Peptic ulcer 1  -Hemorrhagic enterocolitis 2 | 0.204 |
| Other Viral infections** | Total 8 (1.6%)  -Chickenpox 5  -Herpes zoster 3 | Total 13 in 11 cases (4.6%)  -H1N1 6  -Herpes zoster 5  -Rota 2 | 0.023 |
| Second tumors | Total 1 (0.2%)  -2nd ALL after NHL by nine months | Total 2 (0.8%)  -Uterine leiomyoma after 1.6 years from CML  -2nd AML after neuroblastoma by one year | 0.244 |
| Total | 312 (62.2%) | 158 (66.4%) | 0.3 |

SECI indicates South Egypt Cancer Institute; UKK, Cologne University Hospital; CNS, central nervous system; DCL, disturbed conscious level; RTH, radiotherapy; HBV, hepatitis B virus; HCV, hepatitis C virus; ALL, acute lymphoblastic leukemia; NHL, non-Hodgkin lymphoma; CML, chronic myeloid leukemia; and AML, acute myeloid leukemia

*Any patient might have more than one complication, **underestimated in SECI group, ***only complete deafness, mild degrees were underestimated, §Fischer exact test

Total number of children registered in SECI (n=947)

Excluded (n=235)

admitted directly in surgical oncology or radiotherapy department and not admitted or receive any treatment in the pediatric oncology department

Registered in the pediatric oncology department (n=712)

(n= )

Excluded (n=210)

- 102 patients non-malignant diseases
- 39 patients transferred after parents wish to another hospital
- 21 patients died very early after diagnosis
- 18 patients started treatment outside SECI
- 17 patients abandoned after initial suspicion of cancer and before reaching a final diagnosis
- 6 patients abandoned after diagnosis and before start of treatment
- 3 patients had incomplete data
- 2 patients abandoned after referral to initial surgery
- 2 patients had fever of unknown origin referred to the national cancer institute in Cairo for further diagnosis

Analysed (n=502 )

**SUPPLEMENTAL FIGURE 1 Children patients first presented between 2006 and 2010 to South Egypt Cancer Institute (SECI)**

Total number of children registered in the pediatric hematology/oncology department in UKK (n=372)

Excluded (n=134)

- 85 patients started treatment in another hospital
- 33 patients non-malignant diseases
- 9 patients older than 18 years old
- 5 patients were referred to other hospitals by patients' request
- 2 patients died very early after diagnosis within the first day of admission

Analysed (n=238)

**SUPPLEMENTAL FIGURE 2 Children patients first presented between 2006 and 2010 to the pediatric hematology/oncology department in Cologne University Hospital (UKK)**
